# Quercetin Disaggregates Prion Fibrils and Decreases Fibril-Induced Cytotoxicity and Oxidative Stress

**DOI:** 10.3390/pharmaceutics12111081

**Published:** 2020-11-11

**Authors:** Kun-Hua Yu, Cheng-I Lee

**Affiliations:** Department of Biomedical Sciences, Center for Nano Bio-Detections and Center for Innovative Research on Aging Society (CIRAS), National Chung Cheng University, Min-Hsiung, Chia-Yi 62102, Taiwan; ykhuna@gmail.com

**Keywords:** prion, amyloid fibril, quercetin

## Abstract

Transmissible spongiform encephalopathies (TSEs) are fatal neurodegenerative diseases caused by misfolding and aggregation of prion protein (PrP). Previous studies have demonstrated that quercetin can disaggregate some amyloid fibrils, such as amyloid β peptide (Aβ) and α-synuclein. However, the disaggregating ability is unclear in PrP fibrils. In this study, we examined the amyloid fibril-disaggregating activity of quercetin on mouse prion protein (moPrP) and characterized quercetin-bound moPrP fibrils by imaging, proteinase resistance, hemolysis assay, cell viability, and cellular oxidative stress measurements. The results showed that quercetin treatment can disaggregate moPrP fibrils and lead to the formation of the proteinase-sensitive amorphous aggregates. Furthermore, quercetin-bound fibrils can reduce the membrane disruption of erythrocytes. Consequently, quercetin-bound fibrils cause less oxidative stress, and are less cytotoxic to neuroblastoma cells. The role of quercetin is distinct from the typical function of antiamyloidogenic drugs that inhibit the formation of amyloid fibrils. This study provides a solution for the development of antiamyloidogenic therapy.

## 1. Introduction

Amyloid fibrillation is caused by the misfolding of native proteins. Amyloid fibrils have a high tendency to accumulate into insoluble plaques and deposit in tissues and organs. Amyloid fibrils can be found in various amyloidoses, such as Alzheimer’s disease [1], Parkinson’s disease [2], and transmissible spongiform encephalopathies (TSEs) [3] in the brain and neuron. In addition, type-2 diabetes in the pancreas [4] and light chain amyloidosis in the heart, kidney, liver, and peripheral nervous system [5] have been identified as amyloidoses. TSEs have drawn more attention for cross-species transmission since the outbreak of bovine spongiform encephalopathy (BSE) [6]. TSEs are fatal neurodegenerative diseases ascribed to the structural change of prion protein from α-helix-rich isoform (PrP^C^) to β-sheet-rich isoform (PrP^Sc^). Further accumulation of PrP^Sc^ forms insoluble, protease-resistant amyloid fibrils or plaques [3]. Amyloid fibrils can lead to lipid bilayer disruption [7] and cell death [8]. Several studies have shown that PrP^Sc^ interacts with the lipid membrane and causes membrane permeabilization [9,10]. Evidently, cell membrane disruption caused by PrP fibrils leads to cell death.

Variant Creutzfeldt–Jakob disease (vCJD) is one of the prion diseases in humans. It is highly associated with human intake of the BSE pathogen. Therefore, foreign PrP fibrils possess cytotoxicity. PrP^Sc^ is a stable material that is hard to inactivate [11]. According to a recent study on the structure of prion fibrils with a protease-resistant core [12], H111-D144 salt bridge is found in intrafilaments, and each protofilament interacts with hydrophobic force. In addition, each protofilament is stabilized by backbone hydrogen bonds. These inter- and intramolecular interactions make prion fibrils highly stable and difficult to disaggregate. Several materials were found to disaggregate amylin or Aβ amyloid fibrils, including small peptides [13,14,15], nanoparticles [16], and polyphenols [17,18]. Several polyphenol molecules can inhibit Aβ, α-synuclein, or PrP amyloid fibril formation or disaggregate the fibrils, such as epigallocatechin 3-gallate (EGCG) [19], curcumin [20], and nordihydroguaiaretic acid (NDGA) [21].

Oxidative stress occurs in many neurodegenerative diseases [22,23,24]. Flavonoids are a group of polyphenols with high antioxidant ability to react with free radicals and to protect the cells from reactive oxygen species (ROS) [25]. Quercetin is a flavonoid with excellent activity to scavenge free radicals and is one of the dietary antioxidants commonly found in many kinds of food, including apples, onions, green tea, wine, and so on [26]. It is found that quercetin has several beneficial effects in both human cells and rodent models, including cancer prevention [27], cardiovascular protection [28], anti-inflammatory effects [29], and neurodegeneration prevention [30,31].

Previous studies indicate that quercetin can disaggregate Aβ1-42 amyloid fibrils [32,33] and α-synuclein fibrils [34], while the effect of quercetin on PrP fibrils is not clear. Therefore, in this study, we examined the disaggregating activity of quercetin on recombinant mouse PrP (moPrP) fibrils. Furthermore, the effect of quercetin on moPrP fibrils was examined at cellular level, including fibril-caused ROS and cytotoxicity in murine neuroblastoma (N2a) cells.

## 2. Materials and Methods

### 2.1. Expression and Purification of Recombinant moPrP

The pET101/D-TOPO plasmid encoding mouse prion 23-230 (moPrP) was transformed into competent *Escherichia coli* BL21(DE3) cells and over-expressed in inclusion bodies by induction with isopropyl β-d-thiogalactopyranoside. The expressed moPrP proteins were purified on a Ni-Sepharose column based on a previously described method [35] with some modifications [36]. MoPrP was further purified with reversed-phase C4-high performance liquid chromatography (HPLC) column [20]. The purity of the isolated proteins was confirmed by sodium dodecyl sulfate–polyacrylamide gel electrophoresis (SDS–PAGE) and electrospray ionization–mass spectrometry.

### 2.2. Fibril Conversion and Transmission Electron Microscopy

The fibril conversion was performed by incubation of 40 µM moPrP on a shaker rotating at 800 rpm at 37 °C in a buffer solution containing 50 mM 2-(*N*-morpholino)ethanesulfonic acid (MES, pH 6.0) and 2 M guanidine hydrochloride (GdnHCl). The fibril formation was monitored by thioflavin T (ThT), a fluorescence dye recognizing cross-β structure of amyloids. After fibril formation, the fibril samples were dialyzed in H_2_O for further experiments. Quercetin powders were preliminarily dissolved in ethanol as a stock solution of 1 mM. For quercetin-bound fibrils, 2- or 5- equivalent of quercetin were added into moPrP fibrils in 1 mM Tris buffer (pH 7.3) and incubated for 24 h at room temperature. The fibril sample without quercetin treatment is denoted as Fib. The fibrils treated with 2- and 5 equiv. of quercetin are denoted as 2QF and 5QF, respectively. For cell-free assay, 2QF represents 10 µM fibrils treated with 20 µM quercetin. Similarly, 5QF represents 10 µM fibrils treated with 50 µM quercetin. The absorption spectra of quercetin and fibril samples were collected by UV–visible spectrophotometer (SCINCO S-3100, Seoul, Korea). For imaging with transmission electron microscopy (TEM), the fibril samples were stained with 2.6% tungsten phosphoric acid on carbon-coated 200-mesh copper grids. The samples were adsorbed onto the copper grids for 1 min and subsequently washed with phosphate-buffered saline (PBS) and H_2_O. The samples were air-dried before imaging. The images were collected using a transmission electron microscope (JEOL JEM-2100, Tokyo, Japan).

### 2.3. Protease K Digestion

The fibril samples (10 μM) were treated with protease K (PK, 2 ng/µL) at 37 °C in 100 mM Tris (pH 7.5) for 1 h. After the incubation, the PK-treated samples were analyzed by 15% SDS-PAGE. For Western blotting, the proteins were electroblotted onto polyvinylidene difluoride membranes and sequentially incubated with a primary antibody (1 µg/mL SAF-84, epitope moPrP 159–168, Bertin Bioreagent, Montigny le Bretonneux, France) and a secondary antibody (horseradish peroxidase-conjugated anti-mouse IgG). The densitometric quantification of PK-resistant bands was analyzed with ImageJ software, version 1.52a (National Institutes of Health, Bethesda, MD, USA) [37].

### 2.4. Immunostaining and Fluorescence Imaging

Immunofluorescence imaging was performed based on a previously described procedure [38]. Briefly, moPrP fibrils were deposited onto glass slides for 1 h at room temperature and then fixed with 4% formaldehyde for 20 min. After fixation and washing with Tris-buffered saline (TBS), the samples were incubated with the primary antibody 8H4 (6 µg/mL, epitope moPrP 145-179, Abcam, Cambridge, UK) in TBS containing 0.25% Triton X-100 (TBST) and 5% horse serum for 1 h. Subsequently, the samples were stained with a secondary antibody (donkey anti-mouse IgG labeled with Alexa-594) in TBST containing 2% bovine serum albumin for 1 h. In addition, 50 µM ThT was added for the recognition of fibrils. The fluorescence images were collected on a fluorescence microscope (Nikon Eclipse 80i, Tokyo, Japan).

### 2.5. Hemolytic Assay

The hemolytic assay was performed as described previously [20]. In brief, fresh mouse blood was primarily centrifuged at 1000× *g* for 10 min, and the erythrocytes were washed with PBS three times. Afterward, the moPrP fibrils were added into the cell suspensions (1% of hematocrit) for further incubation at 37 °C for 40 min. The fibril-treated erythrocytes were spun down at 1000× *g* for 10 min. Lastly, aliquots of erythrocytes were examined by optical microscopy (Nikon Eclipse 80i, Tokyo, Japan), and the supernatant was collected for absorption measurement.

### 2.6. Cell Viability and ROS Measurements

N2a cells were cultured in Dulbecco’s modified Eagle’s medium (DMEM) supplemented with 10% (*v*/*v*) fetal bovine serum (FBS) and penicillin/streptomycin and 5% CO_2_ at 37 °C. Previously dialyzed 40 µM fibril sample was diluted with DMEM containing 1% FBS to a final concentration of 10 µM before the fibril treatments. Notably, for 2QF and 5QF, 40 µM fibrils were pre-treated with 80 µM and 200 µM quercetin, respectively, prior to the dialysis and following dilution with culture media. N2a cells were cultured in 96-well plates (1 × 10^4^ cells/well) for 24 h. Subsequently, N2a cells were treated with quercetin and/or moPrP fibrils for 24 h. After cell wash with PBS, 10% (*v*/*v*) CCK-8 was added into the cells and incubated for 4 h. The cell viability was determined by the absorbance of formazan recorded at 450 nm using an ELISA reader (Multiskan FC, Thermo Fisher Scientific, Waltham, MA, USA). For ROS measurement, N2a cells cultured in 96-well plates (1 × 10^4^ cells/well) were washed by PBS and then incubated with culture medium containing 1% FBS and 50 µM 2′,7′-dichlorofluorescin diacetate (DCFH–DA) for 1 h. After DCFH–DA incubation, the cells were washed by PBS, and then treated with quercetin and/or fibril samples in culture medium containing 1% FBS for 24 h. In addition, 100 µM hydrogen peroxide was tested as a positive control. The fluorescence emission of DCF at 520 nm was recorded with the excitation wavelength at 485 nm using a microplate reader (FLUOstar Omega, BMG LABTECH, Ortenberg, Germany).

## 3. Results

### 3.1. Effect of Quercetin on Morphology of moPrP Fibrils

**Figure 1:**
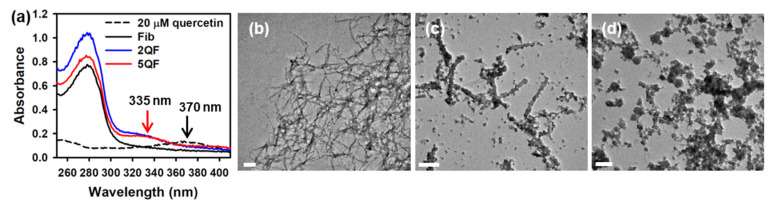
(**a**) UV-Vis absorption spectra of free quercetin, Fib, 2QF, and 5QF. TEM images of (**b**) Fib, (**c**) 2QF, and (**d**) 5QF. The scale bar in TEM images represents 200 nm.

To confirm whether quercetin binds to moPrP fibrils, we measured the absorption spectra of free quercetin and quercetin-bound fibrils as shown in Figure 1a. The major peak of free quercetin is at 370 nm. In quercetin-bound fibrils, the major peak of quercetin shifts to 335 nm. The blue shift of quercetin absorption indicates that quercetin stays in a hydrophobic environment in quercetin-bound fibrils. Moreover, we used TEM to examine how quercetin affects the morphology of moPrP fibrils. As shown in Figure 1b,c, fibrils in Fib are long and straight, but fibrils in 2QF are short and wide. Notably, some amorphous aggregates are shown in 2QF. Significantly, large amorphous aggregates rather than straight fibrils are shown in 5QF (Figure 1d). It is evident that quercetin can disaggregate moPrP fibrils, especially at 5 equiv. dosage.

### 3.2. Effect of Quercetin on Structure and Protease-Resistance of moPrP Fibrils

**Figure 2:**
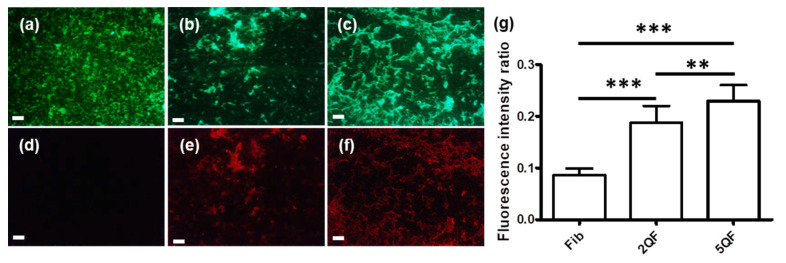
Fluorescence images of fibrils (**a**,**d**) Fib, (**b**,**e**) 2QF, and (**c**,**f**) 5QF shown by ThT (**a**–**c**) and 8H4 (**d**–**f**) staining. The scale bar represents 100 μm. (**g**) Quantitation of the fluorescence intensity ratio of 8H4/ThT. The statistical significance is represented by asterisk (**, *p* < 0.01; ***, *p* < 0.001).

After finding quercetin-induced disaggregation of moPrP fibrils, we looked into the structural difference between three fibril samples by immunofluorescence using the 8H4 antibody that recognizes moPrP 145–179 in the structured C-terminus. The fibrils are well-recognized by ThT images as shown in Figure 2a–c. In Fib, the fibrils were distributed omnipresently and were well recognized by ThT. Clearly, quercetin-bound fibrils have stronger ThT fluorescence due to their high tendency to aggregate. As shown in Figure 2d, Fib is barely recognized by 8H4 due to the buried C-terminal domain within the fibrils. In contrast, as shown in Figure 2e,f, the fluorescence of 8H4 in 2QF and 5QF is enhanced ca. two-fold as quantitated in Figure 2g. The high fluorescence of 8H4 represents the exposure of the C-terminal domain by the treatment of quercetin. Evidently, the addition of quercetin causes structural change in moPrP fibrils.

**Figure 3:**
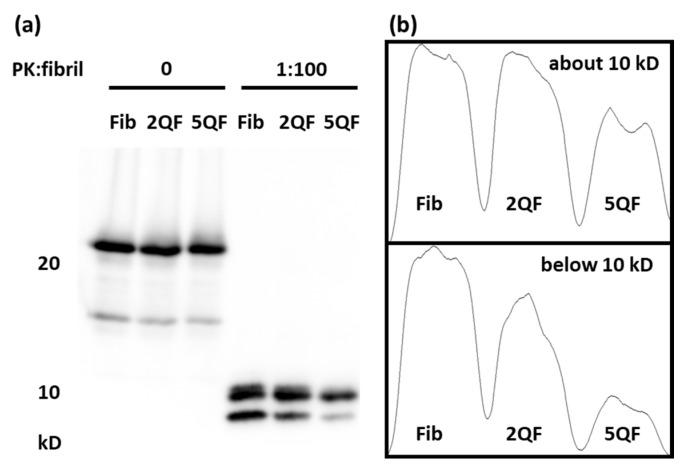
The PK-digested fragments shown in (**a**) immunoblotting by antibody SAF-84. (**b**) Densitometric quantification of PK-resistant fragments. The densitometric patterns in the top and the bottom represents the fragments locate at about 10 kD and below 10 kD, respectively.

A specific characteristic of PrP^Sc^ is its protease resistance. Therefore, we treated moPrP fibrils with PK and analyzed the PK-resistant bands on SDS-PAGE as shown in Figure 3a. Two PK-resistant bands are clearly shown in the low-molecular weight region including one at ~10 kDa and one below 10 kDa. The densitometric quantification shown in Figure 3b indicates that the PK-resistant bands are slightly weakened after the addition of 2 equiv. of quercetin. Furthermore, they are greatly weakened after the addition of 5 equiv. of quercetin, especially for the PK-resistant fragment <10 kDa. These results unambiguously indicate that quercetin modifies the structure of morPrP fibrils and turns moPrP fibrils into partial protease-sensitive.

### 3.3. Effect of Quercetin-Bound moPrP Fibrils on Hemolysis

**Figure 4:**
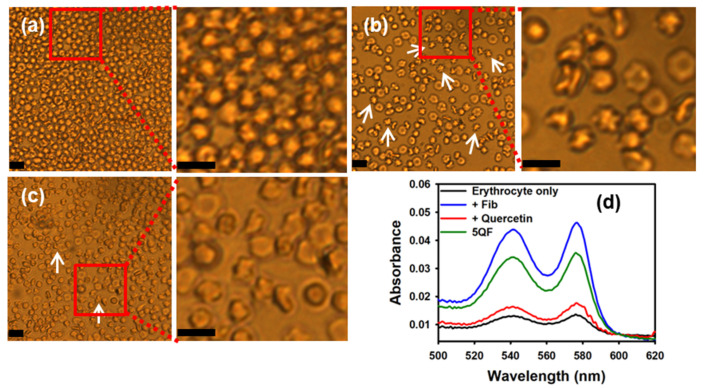
Hemolytic assays of mouse erythrocytes (**a**) without treatment, (**b**) treated with Fib, (**c**) treated with 5QF. In each sample, part of the field is enlarged for clarity. Part of the shrunken cells are shown by arrows in (**b**,**c**). The scale bar represents 10 μm. (**d**) The quantitative comparison of oxy-hemoglobin escaped from erythrocytes monitored by absorption spectroscopy.

Our previous study indicates that the membrane of mouse erythrocytes can be disrupted when they are treated with moPrP fibrils [20]. To examine the effect of quercetin at the cellular level, we examined the morphology of mouse erythrocytes after treatment with moPrP fibrils or quercetin-bound fibrils. The morphology of normal erythrocytes is a biconcave disk in PBS as shown in Figure 4a. When erythrocytes are treated with Fib, as shown in Figure 4b, many of the cells are shrunken. Comparatively, treatment with 5QF causes weak disruption as shown in Figure 4c. The hemoglobin escapes from erythrocytes when the cell membranes get disrupted. To determine the escaped hemoglobin quantitatively, we measured the absorption of oxy-hemoglobin released from erythrocytes at 540 and 576 nm [39]. As shown in Figure 4d, strong absorbance at 540 and 576 nm indicates that Fib treatment causes a significant amount of oxy-hemoglobin escaping from erythrocytes. 5QF treatment causes relatively less oxy-hemoglobin to escape from erythrocytes. 

### 3.4. Effect of Quercetin on moPrP Fibril Induced Cytotoxicity and ROS Level of N2a Cells

**Figure 5:**
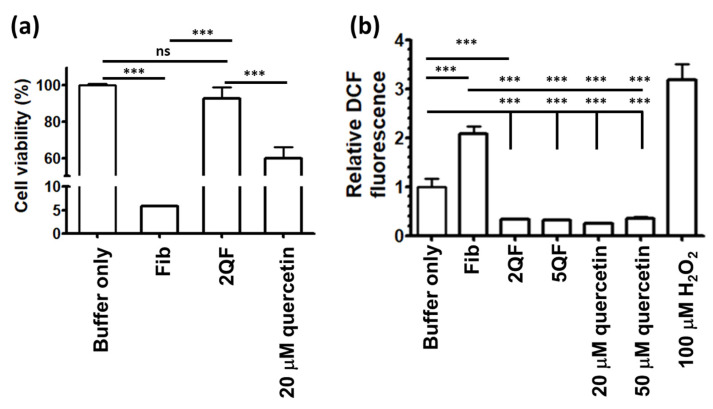
(**a**) Viability of neuroblastoma (N2a) cells treated with buffer (control), Fib, 2QF, and 20 µM quercetin. (**b**) ROS level of N2a cells after treatment with fibrils (Fib, 2QF, or 5QF) or quercetin (20 or 50 µM) determined by intracellular DCF assay. The statistical significance is represented by asterisk (ns, not significant; ***, *p* < 0.001).

To determine the cytotoxicity of moPrP fibrils, we measured the viability of N2a cells after incubation with Fib or 2QF as shown in Figure 5a. Treatment with free quercetin causes about 40% cell death due to the well-known anti-cancer activity of quercetin [26,40]. Therefore, to avoid cytotoxicity induced by free quercetin, the fibril samples were dialyzed before the treatment. Fib is highly cytotoxic, as only 5% of the cells survived. In contrast, 2QF is not cytotoxic. Our previous study indicates that moPrP fibrils can induce oxidative stress in N2a cells [20]. Therefore, we measured the oxidative stress level of N2a cells after treatment with Fib, 2QF, and 5QF by DCF assay. As shown in Figure 5b, in the comparison with buffer treatment, the ROS level is increased to ca. two-fold and three-fold when treating N2a cells with moPrP fibrils and with hydrogen peroxide as a positive control, respectively. Compared with Fib treatment, the ROS level is evidently decreased to one-fifth after incubation with 2QF, 5QF, or quercetin. In Figure 5b, the ROS levels of quercetin treatments are noticeably decreased ascribed to ROS-scavenging ability of quercetin. It is found in several studies that antioxidants can reduce ROS level in cells [41,42]. Similarly, quercetin plays the role of the antioxidant to reduce ROS level in N2a cells. However, quercetin causes cytotoxicity as shown in Figure 5a. In the cellular metabolism, free quercetin is converted to cytotoxic quinone in N2a cells [43]. The cytotoxicity of quercetin is abolished when quercetin is bound to fibrils. Quercetin-bound fibrils significantly decrease the cytotoxicity comparing to treatments of fibril only or quercetin only in N2a cells. These findings indicated that quercetin binding decreases the fibril caused cytotoxicity ascribed to the effect of fibril disaggregation and ROS-scavenging activity. 

## 4. Discussion

Although a compound can disaggregate one kind of amyloid fibrils, this may not apply to the other kinds of amyloid fibrils. For example, NDGA can disaggregate α-synuclein fibrils [44] but cannot disaggregate β-amyloid fibrils [45]. In this study, we found that quercetin can disaggregate prion fibrils. In the process of disaggregation, we observed the structural change of prion fibrils and the loosened fibrils accumulating to form amorphous aggregates. These amorphous aggregates still contain cross-β-sheet structure so that they can be recognized by ThT. However, the C-terminal structure is loosened so that it can be recognized by 8H4 antibody and sensitive for protease K.

The short fibrils are highly cytotoxic [46]. A previous study has found that the amyloid fibrils of PrP106-126 disrupt membrane bilayers. Interestingly, after treatment of PrP106-126 fibrils with bacoside A, the fibrils accumulate into large aggregates and reduce the permeation of membrane bilayers [47]. These findings indicate that the membrane disruption is stronger by small fibrils than by large aggregates. A previous study indicates that the prion fibrils damage the membrane, resulting in increased membrane permeability [48]. The results of ThT fluorescence imaging combined with this hemolysis assay show that it is likely that the moPrP fibrils induce membrane disruption by pore formation, resulting in hemoglobin escape. Quercetin binding can alternate the morphology of moPrP fibrils, resulting in the formation of large aggregates. Therefore, quercetin binding can weaken the membrane damage and reduce escaped hemoglobin.

It is well known that amyloid fibrils cause ROS production [49,50,51]. It is not clear why moPrP fibrils induced ROS production in N2a cells. One possible reason is that plasma membranes are disrupted by moPrP fibrils, resulting in calcium homeostasis dysregulation [52]. The concentration of Ca^2+^ is very low in cytosol (about 100 nM) and high in extracellular space (about 2 mM) [53]. When the plasma membrane is damaged, Ca^2+^ flux would enter cytosol and induce activation of protein kinase C (PKC) which phosphorylates p47-phox and follows assembly of NADPH oxidase 2 (NOX2) complex at plasma membranes. As result, NOX2 generates a large amount ROS [54,55]. Low ROS production in N2a cells caused by quercetin-bound fibrils could be caused by reduced plasma membrane damage. 

## 5. Conclusions

In this study, we have identified the binding of quercetin to moPrP fibrils and the disaggregation of quercetin-bound fibrils. The quercetin binding loosens the structure of C-terminal moPrP in quercetin-bound fibrils, resulting in the sensitivity of the fibrils to PK. Cellular assays indicate that quercetin binding reduces fibril-induced cellular ROS generation, resulting in abolished cytotoxicity. Based on these findings, we believe that quercetin can potentially reduce neuron damage from TSEs.

## References

[1] Selkoe D.J., Hardy J. (2016). The amyloid hypothesis of Alzheimer’s disease at 25 years. EMBO Mol. Med..

[2] Goedert M., Spillantini M.G., Del Tredici K., Braak H. (2013). 100 years of Lewy pathology. Nat. Rev. Neurol..

[3] Prusiner S.B. (1998). Prions. Proc. Natl. Acad. Sci. USA.

[4] Cooper G.J., Willis A.C., Clark A., Turner R.C., Sim R.B., Reid K.B. (1987). Purification and characterization of a peptide from amyloid-rich pancreases of type 2 diabetic patients. Proc. Natl. Acad. Sci. USA.

[5] Desport E., Bridoux F., Sirac C., Delbes S., Bender S., Fernandez B., Quellard N., Lacombe C., Goujon J.M., Lavergne D. (2012). Al amyloidosis. Orphanet J. Rare Dis..

[6] Brown P., Will R.G., Bradley R., Asher D.M., Detwiler L. (2001). Bovine spongiform encephalopathy and variant Creutzfeldt-Jakob disease: Background, evolution, and current concerns. Emerg. Infect. Dis..

[7] Milanesi L., Sheynis T., Xue W.F., Orlova E.V., Hellewell A.L., Jelinek R., Hewitt E.W., Radford S.E., Saibil H.R. (2012). Direct three-dimensional visualization of membrane disruption by amyloid fibrils. Proc. Natl. Acad. Sci. USA.

[8] Novitskaya V., Bocharova O.V., Bronstein I., Baskakov I.V. (2006). Amyloid fibrils of mammalian prion protein are highly toxic to cultured cells and primary neurons. J. Biol. Chem..

[9] Ambadi Thody S., Mathew M.K., Udgaonkar J.B. (2018). Mechanism of aggregation and membrane interactions of mammalian prion protein. Biochim. Biophys. Acta Biomembr..

[10] Caughey B., Baron G.S., Chesebro B., Jeffrey M. (2009). Getting a grip on prions: Oligomers, amyloids, and pathological membrane interactions. Annu. Rev. Biochem..

[11] Rutala W.A., Weber D.J., Society for Healthcare Epidemiology of America (2010). Guideline for disinfection and sterilization of prion-contaminated medical instruments. Infect. Control Hosp. Epidemiol..

[12] Glynn C., Sawaya M.R., Ge P., Gallagher-Jones M., Short C.W., Bowman R., Apostol M., Zhou Z.H., Eisenberg D.S., Rodriguez J.A. (2020). Cryo-EM structure of a human prion fibril with a hydrophobic, protease-resistant core. Nat. Struct. Mol. Biol..

[13] Paul A., Kalita S., Kalita S., Sukumar P., Mandal B. (2017). Disaggregation of amylin aggregate by novel conformationally restricted aminobenzoic acid containing alpha/beta and alpha/gamma hybrid peptidomimetics. Sci. Rep..

[14] Soto C., Sigurdsson E.M., Morelli L., Kumar R.A., Castano E.M., Frangione B. (1998). Beta-sheet breaker peptides inhibit fibrillogenesis in a rat brain model of amyloidosis: Implications for Alzheimer’s therapy. Nat. Med..

[15] Bolarinwa O., Li C., Khadka N., Li Q., Wang Y., Pan J., Cai J. (2020). γ-AApeptides-based small molecule ligands that disaggregate human islet amyloid polypeptide. Sci. Rep..

[16] Jha A., Ghormade V., Kolge H., Paknikar K.M. (2019). Dual effect of chitosan-based nanoparticles on the inhibition of beta-amyloid peptide aggregation and disintegration of the preformed fibrils. J. Mater. Chem. B.

[17] Williams P., Sorribas A., Howes M.J. (2011). Natural products as a source of Alzheimer’s drug leads. Nat. Prod. Rep..

[18] Ishida K., Yamamoto M., Misawa K., Nishimura H., Misawa K., Ota N., Shimotoyodome A. (2020). Coffee polyphenols prevent cognitive dysfunction and suppress amyloid beta plaques in APP/PS2 transgenic mouse. Neurosci. Res..

[19] Andrich K., Bieschke J. (2015). The effect of (-)-epigallo-catechin-(3)-gallate on amyloidogenic proteins suggests a common mechanism. Adv. Exp. Med. Biol..

[20] Lin C.F., Yu K.H., Jheng C.P., Chung R., Lee C.I. (2013). Curcumin reduces amyloid fibrillation of prion protein and decreases reactive oxidative stress. Pathogens.

[21] Freyssin A., Page G., Fauconneau B., Rioux Bilan A. (2018). Natural polyphenols effects on protein aggregates in Alzheimer’s and Parkinson’s prion-like diseases. Neural Regener. Res..

[22] Bossy-Wetzel E., Schwarzenbacher R., Lipton S.A. (2004). Molecular pathways to neurodegeneration. Nat. Med..

[23] Milhavet O., Lehmann S. (2002). Oxidative stress and the prion protein in transmissible spongiform encephalopathies. Brain Res. Rev..

[24] Singh A., Kukreti R., Saso L., Kukreti S. (2019). Oxidative Stress: A key modulator in neurodegenerative diseases. Molecules.

[25] Nijveldt R.J., van Nood E., van Hoorn D.E., Boelens P.G., van Norren K., van Leeuwen P.A. (2001). Flavonoids: A review of probable mechanisms of action and potential applications. Am. J. Clin. Nutr..

[26] Boots A.W., Haenen G.R., Bast A. (2008). Health effects of quercetin: From antioxidant to nutraceutical. Eur. J. Pharmacol..

[27] Murakami A., Ashida H., Terao J. (2008). Multitargeted cancer prevention by quercetin. Cancer Lett..

[28] Tian H., Liu Q., Qin S., Zong C., Zhang Y., Yao S., Yang N., Guan T., Guo S. (2017). Synthesis and cardiovascular protective effects of quercetin 7-O-sialic acid. J. Cell. Mol. Med..

[29] Min Y.D., Choi C.H., Bark H., Son H.Y., Park H.H., Lee S., Park J.W., Park E.K., Shin H.I., Kim S.H. (2007). Quercetin inhibits expression of inflammatory cytokines through attenuation of NF-kappa B and p38 MAPK in HMC-1 human mast cell line. Inflamm. Res..

[30] Choi G.N., Kim J.H., Kwak J.H., Jeong C.H., Jeong H.R., Lee U., Heo H.J. (2012). Effect of quercetin on learning and memory performance in ICR mice under neurotoxic trimethyltin exposure. Food Chem..

[31] Sabogal-Guaqueta A.M., Munoz-Manco J.I., Ramirez-Pineda J.R., Lamprea-Rodriguez M., Osorio E., Cardona-Gomez G.P. (2015). The flavonoid quercetin ameliorates Alzheimer’s disease pathology and protects cognitive and emotional function in aged triple transgenic Alzheimer’s disease model mice. Neuropharmacology.

[32] Ono K., Yoshiike Y., Takashima A., Hasegawa K., Naiki H., Yamada M. (2003). Potent anti-amyloidogenic and fibril-destabilizing effects of polyphenols in vitro: Implications for the prevention and therapeutics of Alzheimer’s disease. J. Neurochem..

[33] Jimenez-Aliaga K., Bermejo-Bescos P., Benedi J., Martin-Aragon S. (2011). Quercetin and rutin exhibit antiamyloidogenic and fibril-disaggregating effects in vitro and potent antioxidant activity in APPswe cells. Life Sci..

[34] Zhu M., Han S., Fink A.L. (2013). Oxidized quercetin inhibits alpha-synuclein fibrillization. Biochim. Biophys. Acta.

[35] Bocharova O.V., Breydo L., Parfenov A.S., Salnikov V.V., Baskakov I.V. (2005). In vitro conversion of full-length mammalian prion protein produces amyloid form with physical properties of PrP(Sc). J. Mol. Biol..

[36] Lin S.J., Yu K.H., Wu J.R., Lee C.F., Jheng C.P., Chen H.R., Lee C.I. (2013). Liberation of GPI-anchored prion from phospholipids accelerates amyloidogenic conversion. Int. J. Mol. Sci..

[37] Schneider C.A., Rasband W.S., Eliceiri K.W. (2012). NIH Image to ImageJ: 25 years of image analysis. Nat. Methods.

[38] Novitskaya V., Makarava N., Bellon A., Bocharova O.V., Bronstein I.B., Williamson R.A., Baskakov I.V. (2006). Probing the conformation of the prion protein within a single amyloid fibril using a novel immunoconformational assay. J. Biol. Chem..

[39] Haurowitz F. (1951). Hemoglobin, anhydro-hemoglobin, and oxyhemoglobin. J. Biol. Chem..

[40] Gibellini L., Pinti M., Nasi M., Montagna J.P., De Biasi S., Roat E., Bertoncelli L., Cooper E.L., Cossarizza A. (2011). Quercetin and cancer chemoprevention. Evid. Based Complementary Altern. Med.

[41] Sayin V.I., Ibrahim M.X., Larsson E., Nilsson J.A., Lindahl P., Bergo M.O. (2014). Antioxidants accelerate lung cancer progression in mice. Sci. Transl. Med..

[42] Sathya S., Shanmuganathan B., Balasubramaniam B., Balamurugan K., Devi K.P. (2020). Phytol loaded PLGA nanoparticles regulate the expression of Alzheimer’s related genes and neuronal apoptosis against amyloid-beta induced toxicity in Neuro-2a cells and transgenic Caenorhabditis elegans. Food Chem. Toxicol..

[43] Mukai R., Kawabata K., Otsuka S., Ishisaka A., Kawai Y., Ji Z.S., Tsuboi H., Terao J. (2012). Effect of quercetin and its glucuronide metabolite upon 6-hydroxydopamine-induced oxidative damage in Neuro-2a cells. Free Radic. Res..

[44] Haney C.M., Cleveland C.L., Wissner R.F., Owei L., Robustelli J., Daniels M.J., Canyurt M., Rodriguez P., Ischiropoulos H., Baumgart T. (2017). Site-specific fluorescence polarization for studying the disaggregation of alpha-synuclein fibrils by small molecules. Biochemistry.

[45] Moss M.A., Varvel N.H., Nichols M.R., Reed D.K., Rosenberry T.L. (2004). Nordihydroguaiaretic acid does not disaggregate beta-amyloid(1-40) protofibrils but does inhibit growth arising from direct protofibril association. Mol. Pharmacol..

[46] Xue W.F., Hellewell A.L., Gosal W.S., Homans S.W., Hewitt E.W., Radford S.E. (2009). Fibril fragmentation enhances amyloid cytotoxicity. J. Biol. Chem..

[47] Malishev R., Nandi S., Kolusheva S., Shaham-Niv S., Gazit E., Jelinek R. (2016). Bacoside-A, an anti-amyloid natural substance, inhibits membrane disruption by the amyloidogenic determinant of prion protein through accelerating fibril formation. Biochim. Biophys. Acta.

[48] Singh J., Sabareesan A.T., Mathew M.K., Udgaonkar J.B. (2012). Development of the structural core and of conformational heterogeneity during the conversion of oligomers of the mouse prion protein to worm-like amyloid fibrils. J. Mol. Biol..

[49] Cheignon C., Tomas M., Bonnefont-Rousselot D., Faller P., Hureau C., Collin F. (2018). Oxidative stress and the amyloid beta peptide in Alzheimer’s disease. Redox Biol..

[50] Jen H.I., Lin Z.Y., Guo J.X., Lee C.I. (2020). The effects of divalent cation-chelated prion fibrils on the immune response of EOC 13.31 microglia cells. Cells.

[51] McWilliams-Koeppen H.P., Foster J.S., Hackenbrack N., Ramirez-Alvarado M., Donohoe D., Williams A., Macy S., Wooliver C., Wortham D., Morrell-Falvey J. (2015). Light chain amyloid fibrils cause metabolic dysfunction in human cardiomyocytes. PLoS ONE.

[52] Demuro A., Mina E., Kayed R., Milton S.C., Parker I., Glabe C.G. (2005). Calcium dysregulation and membrane disruption as a ubiquitous neurotoxic mechanism of soluble amyloid oligomers. J. Biol. Chem..

[53] Clapham D.E. (2007). Calcium signaling. Cell.

[54] Gorlach A., Bertram K., Hudecova S., Krizanova O. (2015). Calcium and ROS: A mutual interplay. Redox Biol..

[55] Brennan-Minnella A.M., Won S.J., Swanson R.A. (2015). NADPH oxidase-2: Linking glucose, acidosis, and excitotoxicity in stroke. Antioxid. Redox Signal..

